# Detection of Anomalous Diffusion with Deep Residual Networks

**DOI:** 10.3390/e23060649

**Published:** 2021-05-22

**Authors:** Miłosz Gajowczyk, Janusz Szwabiński

**Affiliations:** Faculty of Pure and Applied Mathematics, Hugo Steinhaus Center, Wrocław University of Science and Technology, 50-370 Wrocław, Poland; gajowczyk.milosz@gmail.com

**Keywords:** SPT, anomalous diffusion, machine learning classification, deep learning, residual neural networks

## Abstract

Identification of the diffusion type of molecules in living cells is crucial to deduct their driving forces and hence to get insight into the characteristics of the cells. In this paper, deep residual networks have been used to classify the trajectories of molecules. We started from the well known ResNet architecture, developed for image classification, and carried out a series of numerical experiments to adapt it to detection of diffusion modes. We managed to find a model that has a better accuracy than the initial network, but contains only a small fraction of its parameters. The reduced size significantly shortened the training time of the model. Moreover, the resulting network has less tendency to overfitting and generalizes better to unseen data.

## 1. Introduction

Recent advances in single particle tracking (SPT) [1,2,3,4] have allowed to observe single molecules in living cells with remarkable spatio-temporal resolution. Monitoring the details of molecules’ diffusion has become the key method for investigation of their complex environments.

The data collected in SPT experiments often reveal deviations from the Brownian motion [5], i.e., the normal diffusion governed by the Fick’s laws [6] and characterized by a linear time-dependence of the mean square displacement (MSD) of the molecules. Those deviations are referred to as anomalous diffusion, a field intensively studied in the physical community [7,8,9,10]. Since Richardson found a cubic scaling of MSD for particles in turbulent flows [11], anomalous diffusion was observed in many processes including tracer particles in living cells [12,13,14], transport on fractal geometries [15], charge carrier transport in amorphous semiconductors [16], quantum optics [17], bacterial motion [18], foraging of animals [19], human travel patterns [20] and trends in financial markets [21]. Depending on the type of nonlinearity, the anomalous diffusion is further divided into sub- and superdiffusion—two categories corresponding to sub- and superlinear MSD, respectively.

Several analytical approaches have already been attempted to analyze mobility patterns of molecules. The most popular one is based on the mean square displacement [7,22,23,24,25]. The appeal of this method lies in its relative simplicity. However, it is known to have several limitations due to the finite precision of SPT setups [7,22,26,27] and the lack of significant statistics (short trajectories and/or very few ones). To overcome these problems, several other analytic methods have been proposed [27,28,29,30,31,32,33,34,35,36,37,38]. Most of them simply replace MSD by other features calculated from trajectories (e.g., radius of gyration [28] or velocity autocorrelation function [39]).

In the last few years, classification of diffusion modes utilizing machine learning (ML) algorithms is gaining on popularity. Bayesian approach [40,41,42], random forests [43,44,45,46,47], gradient boosting [44,45,46,47], neural networks [48], and deep neural networks [44,49,50,51] have already been used in an attempt to either just classify the trajectories or to extract quantitative information about them (e.g., the anomalous exponent [45,49,51]). The ML approach seems to be more powerful than the analytical one. However, the latter usually offers a deeper insight into the underlying processes governing the dynamics of molecules.

Despite the enormous progress in both the analytical and ML methods, the analysis of SPT data remains challenging. The classification results produced by different methods often do not agree with each other [27,38,46,47]. The reasons are similar to the ones limiting the applicability of MSD: localization errors, short trajectories, or irregular sampling. Thus, there is still need for new robust methods for anomalous diffusion. To catalog the already existing approaches, to assess their usability and to trigger the search for new ones, a challenge (called AnDi challenge) was launched last year by a team of international scientists [52].

In this paper, we are going to present a novel approach to anomalous diffusion based on deep residual networks (ResNets) [53]. In general, deep learning is quite interesting from the perspective of an end user, since it is able to extract features from raw data automatically, without any intervention by a human expert [54]. We already tested the applicability of convolutional neural networks (CNN) to SPT data [44]. They turned out to be very accurate. However, their architecture was quite complicated and the training times (including an automatic search for an optimal model) were of the order of days. Moreover, the resulting network had problems with the generalization to data coming from sources different than the ones used to generate the training set. Residual networks are a class of CNNs able to cure most of the problems the original CNN architecture is facing (i.e., vanishing and/or exploding gradients, saturiation of accuracy with increasing depth). They excel in image classification—a ResNet network won the ImageNet Large Scale Visual Recognition Challenge (ILSVRC) in 2015.

We will start from the smallest of the residual architectures, i.e., ResNet18, and then perform a series of numerical experiments in order to adopt it to characterization of anomalous diffusion. Our strategy for model tuning will be quite simple and focused mainly on the reduction of the parameters of the network. However, it should be noted here that there exist already sophisticated methods for designing small models with good performance [55,56,57,58]. The resulting network will then be applied to the G protein-coupled receptors and G proteins data set, already analyzed in Refs. [38,46,47]. Although our method is not a direct response to the AnDi Challenge [52] (e.g., we use different diffusion models for training), it is consistent with its goal to search for new robust algorithms for classification.

The paper is structured as follows. In Section 2, we briefly introduce the basics of MSD-based methods, the diffusion models we are interested in as well as the residual networks, which will be used for classification. In Section 3, data sets are briefly discussed. The search for the optimal architecture and the performance of the resulting model are presented in Section 4. The results are concluded in the last section.

## 2. Models and Methods

### 2.1. Traditional Analysis

A typical SPT experiment yields a series of coordinates (2D or 3D) over time for every observed particle. Those series have to be analyzed in order to find a relationship between the individual trajectories and the characteristics of the system at hand [59]. Typically, the first step of the analysis is the detection of the type of diffusion encoded in the trajectories.

The most common approach to classification of diffusion is based on the mean-square displacement (MSD) of particles [7,22,23,24,25]. The recorded time series is evaluated in terms of the time averaged MSD (TAMSD),

(1) $$\bar{\delta_{t}^{2}\left(\Delta \right)}=\frac{1}{t-\Delta }\int_{0}^{\infty }{\left[x\left(t^{\prime }+\Delta \right)-x\left(t^{\prime }\right)\right]}^{2}dt^{\prime }$$

where x(t) is the position of the particle at time *t* and Δ is the time lag separating the consequtive positions of the particle. Typically, $\bar{\delta_{t}^{2}\left(\Delta \right)}$ is calculated in the limit Δ≪t to obtain good statistics, since the number of positions contributing to the average decreases with the increasing Δ.

The idea behind the MSD-based method is simply to evaluate the experimental MSD curves, i.e., $\bar{\delta_{t}^{2}\left(\Delta \right)}$ as a function of the varying time lag Δ and then to fit them with a theoretical model of the form

(2) $$\bar{\delta_{t}^{2}\left(\Delta \right)}≃K_{\alpha }\Delta^{\alpha },$$

where $K_{\alpha }$ is the generalized diffusion coefficient and α is the so-called anomalous exponent. The value of the latter one is used to discriminate between different diffusion types. The case α=1 corresponds to the normal diffusion (ND), also known as the Brownian motion [5]. In this physical scenario, a particle moves freely in its environment. In other words, it does not meet any obstacles in its path, and it also does not interact with other distant molecules. Any non-Brownian (α≠1) emanation of particle transport is referred to as the anomalous diffusion. A sublinear MSD (α<1) stands for subdiffusion, which is appropriate to represent particles slowed down due to viscoelastic properties of their surroundings [60], particles colliding with obstacles [61,62] or trapped particles [63,64]. A superlinear case (α>1) indicates superdiffusion, which relates to a fast and usually directed motion of particles driven by molecular motors [65].

### 2.2. Choice of Diffusion Models

Many different theoretical models of diffusion may be used for analysis of experimental data (see Ref. [9] for a detailed overview). However, following Refs. [43,44], we decided to consider four models: normal diffusion [5], directed motion (DM) [22,66,67], fractional Brownian motion (FBM) in subdiffusive mode [68], and confined diffusion (CD) [40]. According to Saxton [7], for those basic models of diffusion in 2D, we have:

(3) $$\begin{array}{ccc} \bar{\delta_{ND}^{2}\left(\Delta \right)} & = & 4D\Delta ,\\ \bar{\delta_{FBM}^{2}\left(\Delta \right)} & = & 4D\Delta^{\alpha },\\ \bar{\delta_{DM}^{2}\left(\Delta \right)} & = & 4D\Delta +{\left(v\Delta \right)}^{2},\\ \bar{\delta_{CD}^{2}\left(\Delta \right)} & ≃ & r_{c}^{2}\left[1-A_{1}\exp\left(\frac{-4A_{2}D\Delta }{r_{c}^{2}}\right)\right]. \end{array}$$

Here, *v* is the drift velocity in the directed motion, the constants $A_{1}$ and $A_{2}$ characterize the shape of the confinement, and $r_{c}$ is the confinement radius.

### 2.3. Deep Learning Classification Methods

The above method has become very popular in the SPT community due to its simplicity. It should work flawlessly for pure long trajectories with no localization errors. However, real trajectories usually contain a lot of noise, which makes the fitting of mathematical models to MSD curves challenging, even in the case of normal diffusion [22]. Moreover, many experimental trajectories are short, limiting the evaluation of the MSD curves to just a few time lags. As a consequence, there is a need for methods going beyond MSD to provide a reliable information concerning the trajectories.

In a recent paper [44], we proposed two machine learning methods that outperform the MSD analysis in case of noisy data. The first one is perceived as traditional machine learning and utilizes a set of human-engineered features that should be extracted from trajectories to feed the classifiers (see also Refs. [46,47] for a more extensive analysis). The second one is based on deep neural networks, which constitute the state-of-the-art of the modern machine learning classification. We showed that both methods perform similarly on the synthetic test data. However, the deep learning approach may seem appealing to practitioners from the SPT community because it usually operates on raw trajectories as input data and does not require human intervention to create features for each trajectory. A cascade of multiple layers of nonlinear processing units is used in this case for automatic feature identification, extraction, and transformation [69].

#### 2.3.1. Convolutional Neural Networks

**Figure 1:**
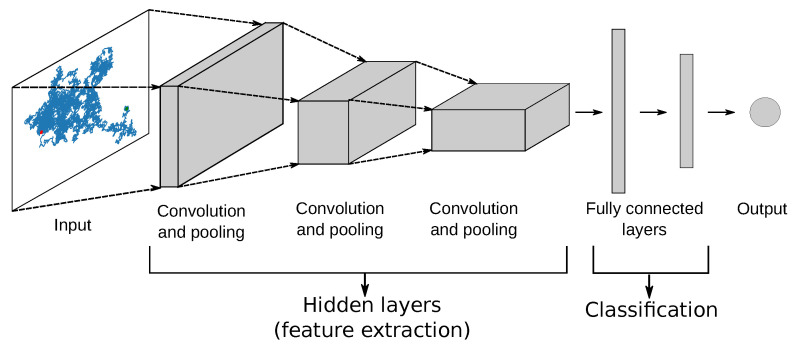
A schematic representation of a CNN network (source: Ref. [44]).

Convolutional neural networks (CNN) were used in Ref. [44] for classification purposes. This choice was triggered by the fact that those networks have already been successful in many tasks including time series analysis [70]. A CNN has usually two components. The first one consisting of hidden layers extracts features from raw data. The fully connected part of the network is responsible for classification (see Figure 1 for a schematic representation of a CNN). In order to detect features in the input data, the hidden layers perform a series of convolutions and pooling operations. Each convolution provides its own map of features (a 3D array) by utilizing a filter that is sliding over the input data. The size of the maps is reduced in the pooling elements.

Choosing the right depth of the network is a challenging task. In Ref. [44], we assumed the architecture of the form (see also Ref. [71] for implementation details)

(4) Batch−[Conv−Batch−ReLu]∗N−Dense−ReLu−Dense−Batch−SoftMax,

and then performed a random search in the architecture and hyperparameter space in order to find the optimal model as well as other parameters required to initialize it. Here, Batch is the batch normalization layer, i.e., a layer performing normalization of the data (not explicitly shown in Figure 1). Conv and Dense stand for convolution and dense layers, respectively. ReLu is the abbreviation of the rectified linear unit, which is an activation function filtering out negative values from the output of the preceding layer. Finally, SoftMax is the activation function determining the final output of the classifier. We haven’t used the pooling layers in this model because reducing the spatial size of the 2D trajectories is usually not necessary. The procedure resulted in a network consisting of six convolutional layers and two dense ones.

#### 2.3.2. ResNet Architecture

Although the model resulting from the above procedure performed well on our synthetic data (accuracy at the level of 97%), its architecture was quite complicated and the network itself was relatively deep, resulting in processing times of the order of days on a cluster of 24 CPUs with 50 GB total memory. However, long training times were not the only issue. It is known that with the increasing depth the problem of vanishing/exploding gradients may appear in the training phase of neural networks. Moreover, the training error may increase with the number of layers, resulting in a saturation of accuracy [53].

**Figure 2:**
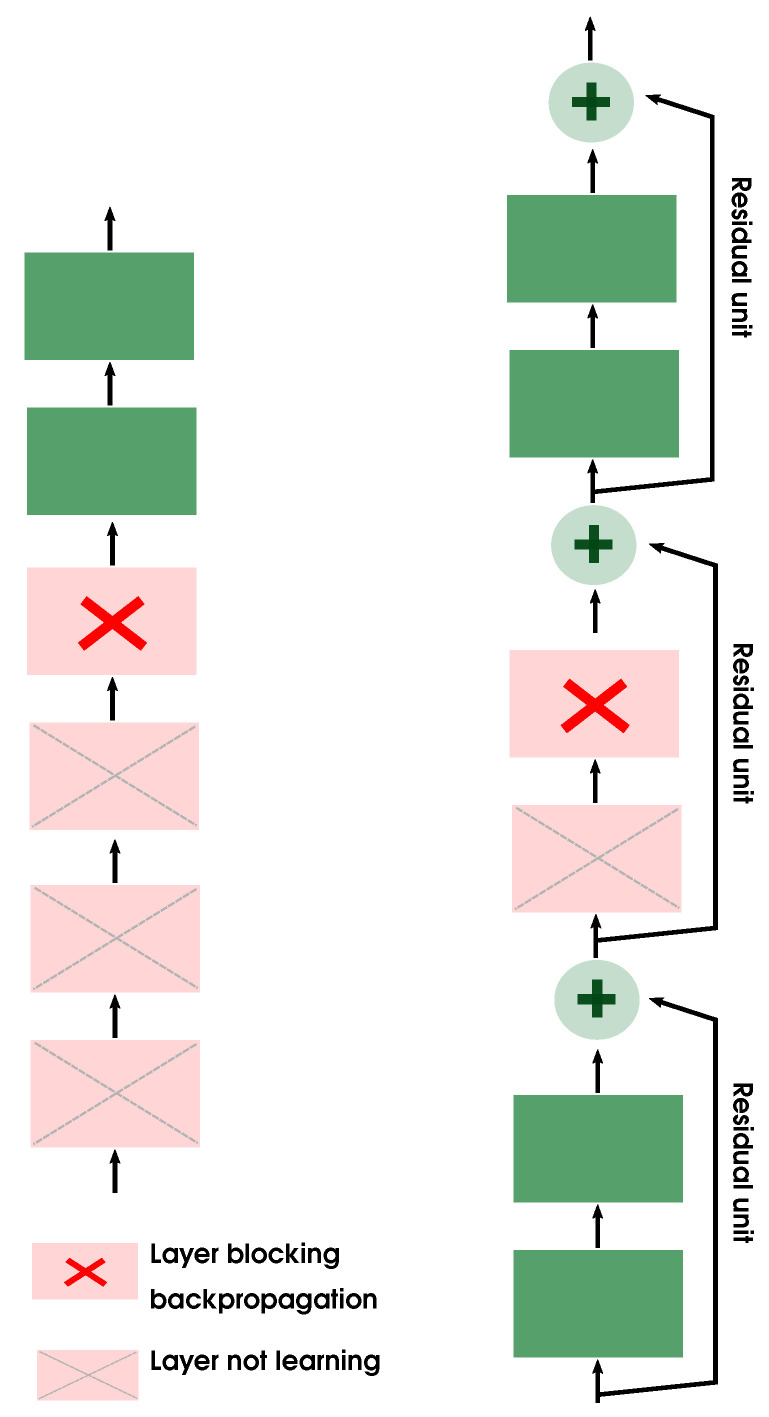
A regular CNN (left) versus a Resnet. Thanks to the skip connections in ResNet, the signal can easily pass a blocking layer in the backpropagation phase.

This is the reason why in this paper we decided to use the residual network (ResNet) [53]. It is a class of CNNs, which utilizes shortcuts (skip connections) to jump over several layers of the networks. Those shortcuts allow the network to make progress even if several layers have stopped learning because there is one blocking the backpropagation (Figure 2).

**Figure 3:**
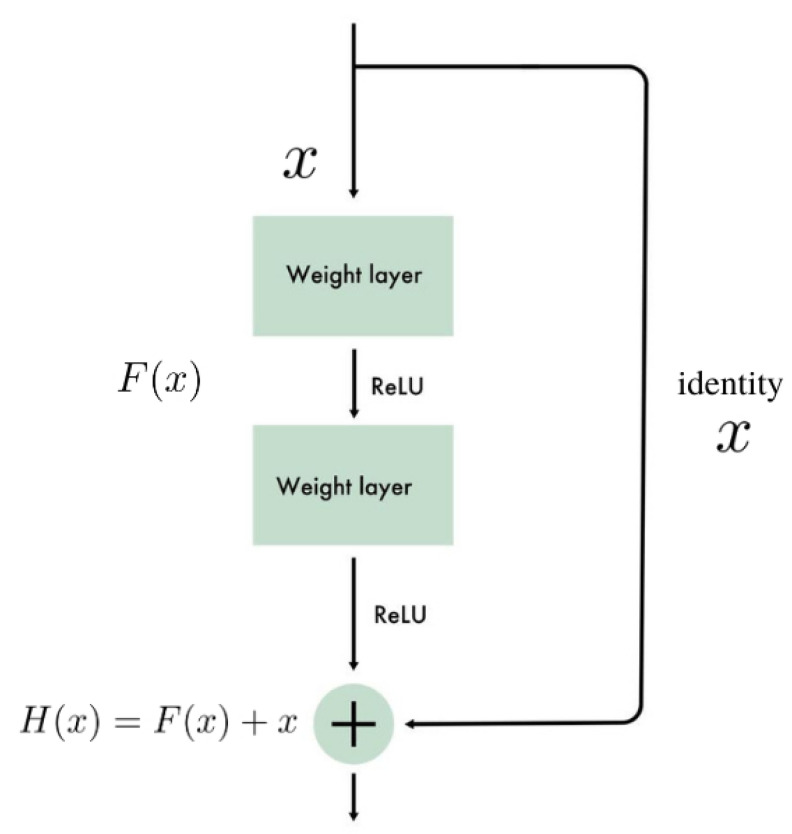
Residual unit in ResNet.

The residual network may be understood as a stack of residual units, where each unit is a small neural network with a skip connection. The outline of the unit is shown in Figure 3. For given input *x*, the desired mapping we want to obtain by learning is H(x). Since the shortcut connection carries out the input layer to the addition operator shown in the figure, the rest of the unit needs only to learn the residual mapping F(x)=H(x)−x. When a regular CNN network is initialized, its weights are close to zero, so the network just outputs values close to zero. After adding the shortcuts, the network initially models the identity function. Therefore, if the target function is close to that function (which is often the case), the training phase will be significantly shorter than in the case of a regular CNN.

**Figure 4:**
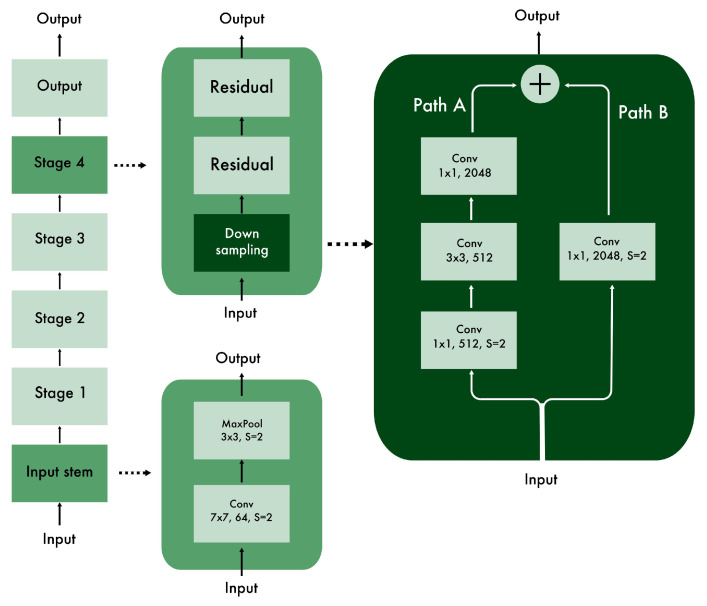
The architecture of ResNet. The downsampling block at the beginning of each stage help to reduce the amount of information in the case of deeper networks (path B is used in this case).

In Figure 4, the actual ResNet architecture is shown. We see that the core of the network is divided into four stages. Each of them contains, in addition to the residual units, a downsampling block. Its role is to reduce the information making its way across the network.

#### 2.3.3. XResNet

In 2018, three modifications to the original ResNet architecture have been proposed under the common name XResNet [72]. Going into their details is beyond the scope of this paper. However, since they are known to have a non-negligible effect on the accuracy of the resulting model in some scenarios, we decided to include them in our search for the optimal architecture.

## 3. Synthetic and Experimental Data

### 3.1. Synthetic Training Data

The main factor limiting the deployment of machine learning to trajectory analysis is the availability of high-quality training data. It should contain a reasonable (i.e., large) amount of input data (trajectories) and corresponding desired output (their diffusion types). Since real data from experiments is not really provable (otherwise we would not need any new classification method), synthetic sets generated with computer simulations of different diffusion models are used for training. An ML algorithm uses the input–output pairs to learn the rules for data processing. Once trained, it is able to use those rules to classify new unseen trajectories.

As already mentioned in Section 2.2, we decided to follow Refs. [43,44] and use four basic models of diffusion to generate the training set of trajectories. The simulation methods will be briefly described in the remaining part of this section.

#### 3.1.1. Normal Diffusion

Although several equivalent methods for simulation of Brownian motion exist, we will follow the approach presented by Michalet [22]. In case of normal diffusion, the probability distribution of the displacement’s norm of a particle is given by the Rayleigh distribution

(5) $$P\left(u\right)=\frac{2u}{4D\Delta t}\exp\left(\frac{-u^{2}}{4D\Delta t}\right),\;u\geq 0,$$

where *u* is the absolute distance traveled by the particle in time Δt. Thus, to simulate a trajectory, we have to randomly choose a start position of a particle and a random direction of the displacement φ and then pick a random step length *u* from the distribution (5). The new position of the particle is calculated,

(6) $$\begin{array}{ccc} x_{new} & = & x_{old}+u\cos\varphi ,\\ y_{new} & = & y_{old}+u\sin\varphi , \end{array}$$

and taken as the starting point for the next move. The whole procedure is repeated till a trajectory of a desired length is generated.

#### 3.1.2. Directed Motion

The simulation algorithm for the Brownian motion may be easily extended to generate a trajectory for diffusion with drift. All we have to do is simply to add a correction to the particle’s position due to its active motion:

(7) $$\begin{array}{ccc} dx_{i} & = & v\Delta t\cos\beta , \end{array}$$

(8) $$\begin{array}{ccc} dy_{i} & = & v\Delta t\sin\beta , \end{array}$$

where *v* is the norm of the drift velocity and β its direction. Once we have the corrections, we add them to the new coordinates:

(9) $$\begin{array}{ccc} x_{new} & = & x_{old}+u\cos\varphi +dx_{i},\\ y_{new} & = & y_{old}+u\sin\varphi +dy_{i}. \end{array}$$

The drift velocity is one of the parameters of the simulation. However, instead of setting its value directly, we will rather use an active-motion-to-diffusion ratio [43]:

(10) $$R=\frac{v^{2}T}{4D},$$

where *T* is the time duration (i.e., the length of the trajectory). In our simulations, we will draw a random value of *R* from a given range and then calculate *v* for given *D* and *T*. In this way, it will be easier to generate similar trajectories with different values of *v* and *D*.

#### 3.1.3. Confined Diffusion

Again, a small modification of the model for normal diffusion is needed to simulate a particle confined inside a reflective circular boundary. We simply divide every step of the simulation into 100 substeps with $\Delta t^{\prime }=\Delta t/100$. Then, a normal diffusion move is carried out in every substep. The new position of the particle after all substeps will be updated only if the distance from the center of the boundary to new coordinates is smaller than the radius $r_{c}$ of the boundary.

Following Wagner et al. [43], we will introduce a boundedness parameter *B*, defined as the area of the smallest ellipse enclosing a normal diffusion trajectory (with no confinement) divided by the area of the confinement,

(11) $$B=\frac{A_{ellipse}}{\pi r_{c}^{2}}≃\frac{DN\Delta t}{r_{c}^{2}}.$$

It will help us to control the level of trapedness of particles in the simulations. *B* will be set randomly for each synthetic trajectory. Based on its value, the radius $r_{c}$ will be calculated for given *D*, *N*, and Δt.

#### 3.1.4. Fractional Brownian Motion

In addition to the confined diffusion, we will also use fractional Brownian motion to simulate the subdiffusive motion. FBM is the solution of the stochastic differential equation

(12) $$dX_{t}^{i}=\sigma dB_{t}^{H,i},i=1,2,$$

where $\sigma =\sqrt{2D}$ is the scale parameter related to the diffusion coefficient *D*, H∈(0,1) is the Hurst parameter and $B_{t}^{H}$ is a continuous-time, zero-mean Gaussian process starting at zero, with the following covariance function

(13) $$E\left(B_{t}^{H}B_{s}^{H}\right)=\frac{1}{2}\left({\left|t\right|}^{2H}+{\left|s\right|}^{2H}-{\left|t-s\right|}^{2H}\right).$$

The Hurst parameter *H* is connected with the anomalous exponent α via the relation

(14) $$H=\frac{\alpha }{2}.$$

Since we want to use FBM for subdiffusion (i.e., α<1) only, the values of *H* will be restricted to the interval (0,1/2) in the simulations.

#### 3.1.5. Creating Noisy Data

Real measurements of particles’ positions are usually altered by noise from different sources including localization errors, vibrations of the sample, electronic noise or errors in the postprocessing phase [73]. Different methods of adding noise to synthetic trajectories are possible. One can, for instance, vary the diffusion coefficient of particles or simply add some disturbance to every point of a trajectory. We will go for the latter method and add normal Gaussian noise with zero mean and standard deviation σ to each simulated position.

To easily generate trajectories characterized by different levels of noise, we will proceed in the following way. We first introduce the signal-to-noise ratio:

(15) $$Q=\left\{\begin{array}{cc} \frac{\sqrt{D\Delta t}}{\sigma } & for\;ND,CD,and\;FBM,\\ \frac{\sqrt{D\Delta t+{\left(v\Delta t\right)}^{2}}}{\sigma } & for\;DM. \end{array}\right.$$

Then, we will randomly set *Q* and use the above formula to determine the standard deviation σ appropriate for given *D*, Δt, and *v*.

#### 3.1.6. Simulation Details

For the sake of comparison, our synthetic data set should resemble all characteristics of the one used in Ref. [44]. To recap, we generated 20,000 trajectories, 5000 for each diffusion type. The time lag between consecutive points within a trajectory was set to Δt=1/30 s, which is a typical value in experimental setups. All other parameters of the diffusion models were chosen randomly from the predefined ranges. Details can be found in Table 1.

The data set was then divided into three subsets: the training set for fitting the machine learning models, the validation set used to estimate prediction errors for model selection and the test set for assessment of the final model. The stratified sampling method [74] was used for that purpose to guarantee a balanced representation of the diffusion modes in the subsets. Their sizes are presented in Table 2.

### 3.2. Real Data

We will apply our classifier to data from a single particle tracking experiment on G protein-coupled receptors and G proteins, already analyzed in Refs. [38,46,47]. The receptors mediate biological effects of many hormones and neourotransmitters and are also important as pharmacological targets [75]. Their signals are transmitted to the cell interior via interactions with G proteins. The analysis of the dynamics of these two types of molecules is extremely interesting because it may shed more light on how the receptors and G proteins meet, interact, and couple.

## 4. Results

The main goal of this work was to find a deep residual network with the simplest possible architecture, which is able to detect types of anomalous diffusion with satisfactory accuracy. In this section, we will first present a series of experiments that allowed us to significantly reduce the number of parameters of the original ResNet architecture. Then, we will apply the resulting model to classify both synthetic and real trajectories. All results were obtained with custom Python codes, available at https://github.com/milySW/NNResearchAPI, accessed on 20 May 2021. PyTorch library [76] was used to build the neural networks.

### 4.1. Finding the Optimal Network Architecture

We performed a series of computer experiments to find a reasonable ResNet architecture. Our goal was to keep the network as small as possible to reduce both the training times and the danger of overfitting. At the same time, we targeted the classification performance on synthetic data beyond the accuracy of 90%.

Before we dive into the results of the most important experiments, we would like to provide one important note. It is usually not worth investing effort and time in more complicated networks for tiny improvements of accuracy because, due to the stochastic nature of the networks, even different instances of the same model may yield slightly different results. Having that in mind, we introduced a (rather arbitrary) threshold equal to 0.2 percentage point as an indicator of improvements worth considering. All changes in accuracy smaller than the threshold were seen as irrelevant.

#### 4.1.1. Impact of XResNet Modifications

Our first attempt was to check if the XResNet modifications [72] to the original architecture are worth considering. We took ResNet18, i.e., the smallest residual network with 18 layers, as the starting point. Results are shown in Table 3. Although the original architecture performs better on the training set, the modified one generalizes better to unseen data (i.e., has higher accuracy on the validation set). This may indicate the tendency of ResNet18 to overfit. The cost we have to pay for the improvement in validation accuracy by 0.34 percentage point is the increase in the number of parameters of the model (by 43,328) and a longer average time needed to complete one epoch (i.e., one cycle through the training data set). Despite the cost, we will keep the modifications in the model and try to reduce the number of parameters by other means.

#### 4.1.2. Depth of Neural Network

The baseline ResNet architecture consists of four stages, each of which is characterized by a different number of kernels that are convolved with the input [53]. However, ResNet was designed for classification of images, which are usually more complex than our trajectories. Thus, it will be interesting to check how a partial removal of those stages impacts the accuracy of the classifier. Results of our experiments are shown in Table 4. We see that reducing the depth of the network leads to a significant decrease in the number of the parameters in the model and improves its accuracy on the validation data.

As expected, one does not need the full depth of the original ResNet architecture to classify the trajectories. Although the number of the parameters for two stages is very tempting, we decided to go further with depth 3 because it gives a slightly better performance.

#### 4.1.3. Dimension and Size of Convolutions

The original Resnet architecture works with 2D objects and uses convolution kernels of size 3×3. It will be interesting to see how the model performs with smaller kernels. Although a 2×2 kernel is theoretically possible, one usually tries to avoid kernels of even sizes due to the lack of a well defined central pixel. Consequently, we will compare only 1×1 kernels with the baseline. As it follows from Table 5, the accuracy of the model declines significantly with the introduction of the smaller kernels.

There is also a possibility of flattening the trajectories to 1D vectors and convolve them with 1×X kernels. We have checked the model for kernels with an odd *X* ranging from 3 to 11. Results are shown in Table 6. As we can see, those changes could slightly improve the performance of the model. Moreover, the size of the model was reduced by 44%. Thus, we will keep 1×5 kernels and work with 1D input for further investigations.

#### 4.1.4. Feature Maps

The number of parameters of the model may also be reduced by limiting its “breadth”, understood here as the number of feature maps (convolution kernels) at each layer. The latter for the *i*-th block is given by the formula:

(16) $$\left\{\begin{array}{c} x_{0}=64,\\ x_{i}=x_{0}\cdot 2^{i-1},\;\;\mathrm{for}\;i\;=\;1,\;2,\;\ldots ,\;n. \end{array}\right.$$

From Table 7, it follows that decreasing $x_{0}$ from 64 to 32 will not significantly decrease the accuracy of the model, but will reduce the number of parameters by a factor of 4. Moreover, the learning process of the network takes noticeably less time.

#### 4.1.5. Additional Features

One of the advantages of deep networks, at least from the perspective of an end user, is the ability to work with raw experimental data. There is no need for human-engineered features as input because the network extracts its own features automatically from the data. While this is true for ResNet architecture as well, in principle, we could augment the input to the model by some additional attributes, including the ones tailor-made to the problem of diffusion.

A set of features with the potential of distinguishing different diffusion modes from each other was presented in Ref. [44]. Here, we would like to check if adding some of those attributes to the model will have a positive impact on accuracy. We decided to use asymmetry, efficiency, fractal dimension, and TAMSD at lag 20 as additional input (see Refs. [43,44] for definitions). For each trajectory, the values of the attributes were added to the network after the raw data went through all convolutional layers and was flattened.

**Figure 5:**
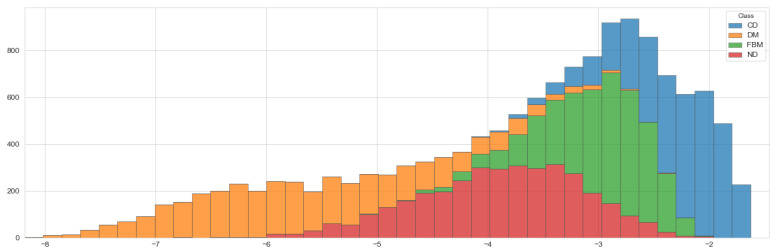
Distribution of asymmetry among trajectories in the synthetic data for different types of diffusion.

Results of this series of experiments are shown in Table 8. Although the network was fed with additional information, its accuracy has not improved. To explain that, let us have a look at the distribution of asymmetry among trajectories in our data set. As it follows from Figure 5, its values for different types of diffusion overlap to some extent. Thus, classifying them based on the information encoded in asymmetry may be challenging. The same holds for the other attributes. Thus, we are not going to include them in our final model.

#### 4.1.6. Impact of Autocorrelation

Following Ref. [77], we decided to check if the autocorrelation function taken as additional input improves the accuracy of the model. We combined the raw trajectories with their autocorrelations calculated at lags 8, 16, and 24 into a single tensor structure and used it as input to the model. Again, this measure did not improve the accuracy (Table 9).

#### 4.1.7. Selective Backprop

One of the interesting techniques to accelerate the training of deep neural networks is the selective backprop [78]. The idea behind this procedure is to prioritize samples with high loss at each iteration. It uses the output of sample’s forward pass in the training phase to decide whether to use that sample to compute gradients and update parameters of the model or to skip immediately to other sample.

We carried out an experiment with two selective backprop scenarios. In the first one, a subset of training data covering 98% of the total loss was chosen for back-propagation. In this way, only 50–60% of trajectories were used in every epoch to update the network. In the second scenario, 50% of the training data were always taken, covering between 94% and 99% of the total loss in each epoch. It turned out that this method indeed shortens the training phase of the network (in particular average epoch time). However, it yields worse performance compared to the model utilizing the whole data set for back-propagation (Table 10).

#### 4.1.8. Choice of Hyperparameters

In the last series of experiments, we tried to find optimal values of some hyperparameters of the model. First, we looked at the cost function. Its choice allows us to control the focus in the training phase. Cross entropy for instance strongly penalizes misclassification, as it grows exponentially while approaching a wrong prediction [79]. Mean squared error (MSE) is usually used for regression problems. It does not punish wrong classifications enough, but rather promotes being close to a desired value. Although the cross entropy is the natural choice in classification tasks, the choice of the cost function seems to have no significant impact on the model’s validation accuracy (Table 11). We kept MSE for shorter training times.

An activation function defines the output of a node for the given input. It usually introduces some nonlinearity to the model. We checked four different functions. Sigmoid [80] is one of the most widely used activation functions today. It nicely mimics the behavior of real neurons; however, it may suffer from vanishing/exploding gradients. ReLU [81] is computationally very cheap, but it is also known to “die” in some situations (weights may update in such a way that the neuron never activates). Leaky ReLU [82] and ELU [83] are modifications of ReLU that mitigate that problem.

According to Table 12, ReLU activation function offers the highest accuracy on the validation set.

The batch size is another important hyperparameter in the model. It defines the number of samples to work through before the model’s internal parameters are updated. Larger batches should allow for more efficient computation, but may not generalize well to unseen data [84]. Small batches, on the other hand, are known to sometimes have problems with arriving at local minima [79].

Results for three different batch sizes are shown in Table 13—512 turned out to be the best one in our model.

#### 4.1.9. Resulting Model

Based on the results of the above experiments, we were able to reduce the number of parameters in the model from 11,220,420 in Resnet18 with XResNet modifications to 399,556. In the same time, the accuracy of the model on validation data increased by 1.33 percentage points.

The architecture of the final model is summarized in Table 14. Besides the already mentioned parameters and hyperparameters, there are two others that have not been discussed yet. The activation threshold is a boolean flag telling the model whether it should automatically estimate the threshold value, above which the neurons become active. In addition, the learning rate is a tuning parameter that determines the step size at each iteration while moving toward a minimum of the loss function. To find its value, we used a finder algorithm proposed in Ref. [85] and implemented in a PyTorch Lightning module [86].

### 4.2. Performance of the Model

**Figure 6:**
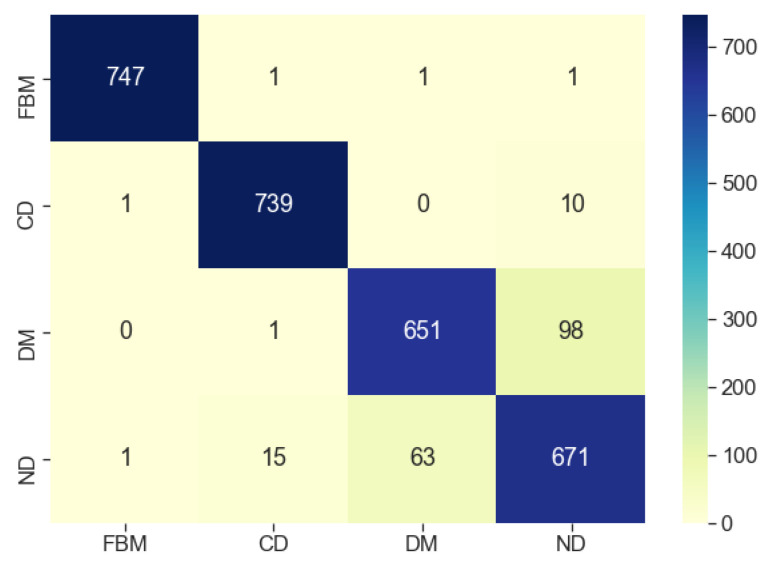
Confusion matrix of the model. Rows correspond to the true labels and columns to the predicted ones.

A test set consisting of 3000 samples (750 for each diffusion type) was used to assess the performance of the final model (see Section 3.1.6 for details). In Figure 6, the confusion matrix of the classifier is shown. By definition, an element $C_{ij}$ of the matrix is equal to the number of observations known to be in class *i* (true labels) and predicted to be in class *j* [87].

The model achieves the best performance for subdiffusion. Only 12 out of 750 trajectories have been wrongly classified in case of FBM and 25 out of 750 in case of CD. The other two modes are more challenging for the classifier. As for DM, 136 trajectories are misclassified, most of them as normal diffusion. The performance for the latter is slightly better—109 trajectories got wrong labels.

In Section 4.1.5, we tried to improve the performance of the model with some additional human-engineered features, which were motivated by the characteristics of diffusion itself. We were not really successful because it turned out that the distributions of those features overlap with each other, particularly for DM and ND, contributing to the confusion of the classifier. We guess that the same holds for features extracted automatically by the ResNet model—they are not specific enough to better distinguish DM from ND.

The confusion matrix may be used to calculate the basic performance metrics of the classifier. They are summarized in Table 15. Accuracy is defined as the number of correct predictions divided by the total number of predictions. Precision is the fraction of correct predictions of a class among all predictions of that class. It indicates how often a classifier is correct if it predicts a given class. Recall is the fraction of correct predictions of a given class over the total number of samples in that class. It measures the number of relevant results within a predicted class. Finally, F1 score is the harmonic mean of precision and recall.

Even though the model has apparently some problems with DM and ND classes, its overall accuracy on test data are high. It returns much more relevant results than the irrelevant ones (high average precision), and it is able to yield most of the relevant results (high average recall). The F1 score simply confirms that.

**Figure 7:**
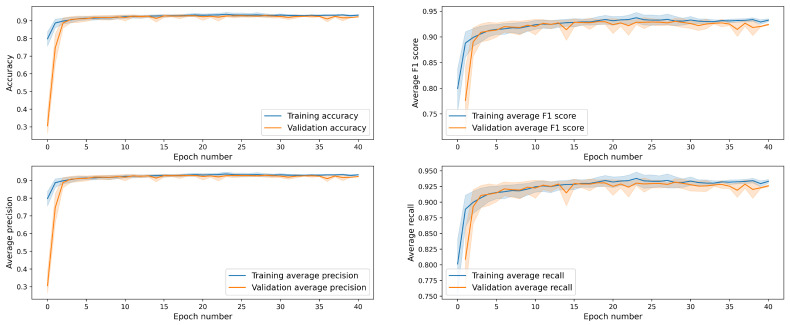
Performance metrics (on validation data) of the model as functions of the training time.

It could be also interesting to check how the performance metrics of the classifier evolve with the training time (i.e., with the number of epochs). The results are presented in Figure 7. To generate the plots, we trained 50 instances of the model and then averaged the metrics. In this way, we could also estimate the 95% confidence levels. We see that all metrics reach a satisfactory level already in the third epoch. Further training improves the performance of the model only slightly.

**Figure 8:**
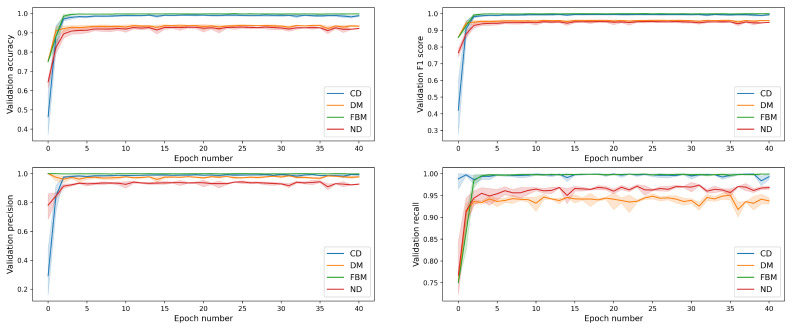
Performance metrics (on validation data) for each diffusion mode as functions of the training time.

The same results, but this time broken down into separate diffusion modes, are shown in Figure 8. The measures for DM and ND are not only smaller than the ones for subdiffusion, but they also fluctuate to a higher extent when we look at values after the early epochs. This is due to the fact that these two classes are often confused with each other.

**Figure 9:**
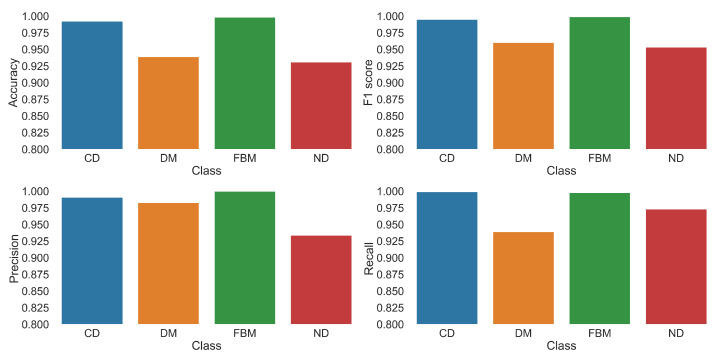
Performance metrics in the best epoch for each diffusion mode.

The metrics for individual classes in the best epoch are shown in Figure 9. Again, we see a small gap between the subdiffusive classes on one hand and the problematic ones (i.e., DM and ND) on the other. However, even in the worst case, the metrics are above 80% indicating a good performance of the classifier.

### 4.3. Classification of Real Data

From the available data on G protein-coupled receptors and G proteins, we took into account only trajectories with at least 50 steps. In this way, the data set was reduced to 1029 G proteins and 1218 receptors. Classification results are shown in Table 16. For the sake of comparison, two other predictions are reported in the table: a gradient boosting method utilizing noisy training data and a set of human-engineered features (reduced Set A trained with noise, see Table 15 in Ref. [47] for details) and a statistical testing procedure based on the maximum distance traveled by the particle (MAX method, see Refs. [38,46] for details).

Despite some differences in the absolute numbers, all three methods classify most of the trajectories as normal diffusion. However, there are significant discrepancies between them in the classification of the remaining time series. While our method labels almost all of them as superdiffusion, the other two ones predict subdiffusion in most of the cases. Unfortunately, the ground-truth for real data are missing and the results cannot be proven. However, it was already pointed out in Ref. [38] that different classification algorithms may provide substantially different results for the same data sets. Averaging of the results from all available methods has been proposed to mitigate the risk of large classification errors.

## 5. Discussion and Conclusions

Identifying the type of motion of particles in living cells is crucial to deduct their driving forces and hence to get insight into the mechano-structural characteristics of the cells. With the development of advanced AI methods in the last decades, there is an increasing interest to use them for that purpose. These methods are expected to outperform the well established statistical approach, in particular for noisy and small data sets.

In this paper, deep residual networks have been used to classify the SPT trajectories. We started from the well-known ResNet architecture [72], which excels in image classification, and carried out a series of numerical experiments to adapt it to detection of diffusion modes. We managed to find a model that has a better accuracy than the initial network, but contains only a small fraction of its parameters (399,556 vs. 11,177,092 in ResNet18, i.e., the smallest among ResNet networks). The reduced number of parameters had a huge positive impact on the training time of the model. Moreover, the resulting network has less tendency to overfitting and generalizes better to unseen data.

The overall accuracy of our model on the synthetic test data with noise is pretty good (90.6%). Breaking down the predictions into individual classes reveals that the model is able to recognize FBM and confined diffusion with a remarkable accuracy (99.6% and 98.53%, respectively). The detection of normal diffusion and directed motion seems to be more challenging and the model mixes up those two categories with each other from time to time.

Regarding the classification of real data, the predictions of our model are a little bit confusing. Compared to two other methods, i.e., a statistical testing procedure based on the maximum distance traveled by the particle [38,46] and gradient boosting methods with a set of tailor-made features characterizing the trajectories [47], it gives a similar fraction of normal diffusion (the majority class) among the trajectories. However, while our model classifies the remaining data as superdiffusion, the other ones assign most of those trajectories to the subdiffusive class. Moreover, it should be mentioned that some other classifiers provide results different from the ones in Table 16 [38,46]. In light of the above, the authors in Ref. [38] suggested taking a mean of the results of all available methods to minimize the risk of large errors. Therefore, there is still need to search for new classification methods for SPT data.

## Figures and Tables

**Table 1:** Parameters of the simulation and their values. All values except Δt were randomly chosen from given ranges.

Parameter	Meaning	Range of Values
Δt	timelag between steps	1/30 [s]
*D*	diffusion coefficient	0.1–20 [μm${}^{2}$/s]
*N*	length of a trajectory	30–600
*B*	boundedness	1–6
*R*	active motion to diffusion ratio	1–17
α	anomalous exponent	0.3–0.7
SNR	signal to noise ratio	1–9

**Table 2:** Partition of the synthetic data set.

Subset Type	FBM	CD	DM	ND	Size	Share
Training	3500	3500	3500	3500	14,000	70%
Validation	750	750	750	750	3000	15%
Test	750	750	750	750	3000	15%

**Table 3:** Impact of the XResNet modifications [72] on the accuracy of the model. Bold indicates the architecture we chose for further investigations.

Architecture	Number of Parameters	Accuracy (Training)	Accuracy (Validation)	Best Epoch	Epoch Time [s]
ResNet	11,177,092	93.16%	90.33%	12	40
**XResNet**	11,220,420	92.19%	90.67%	21	48

**Table 4:** Relationship between the accuracy of the model and its depth. Depth equal to 3 was chosen for further investigations.

Depth	Number of Parameters	Accuracy (Training)	Accuracy (Validation)	Best Epoch	Epoch Time [s]
4	11,220,420	92.19%	90.67%	21	48
**3**	2,823,108	91.32%	91.10%	25	33
2	721,604	90.26%	90.80%	38	28

**Table 5:** Relationship between the size of the 2D convolution kernels and the performance of the model.

Conv. Kernel	Number of Parameters	Accuracy (Training)	Accuracy (Validation)	Best Epoch	Epoch Time [s]
1×1	357,188	75.95%	76.93%	24	9
3×3	2,823,108	91.32%	91.10%	25	33

**Table 6:** Relationship between the size of the 1D convolution kernels and the performance of the model.

Conv. Kernel	Number of Parameters	Accuracy (Training)	Accuracy (Validation)	Best Epoch	Epoch Time [s]
1×3	973,668	92.69%	91.23%	25	39
1×5	1,590,148	92.77%	92.17%	30	24
1×7	2,206,628	90.91%	91.60%	14	28
1×9	2,823,108	93.59%	91.17%	20	30
1×11	3,439,588	92.71%	91.70%	15	32

**Table 7:** Relationship between the number of feature maps and the accuracy of the model.

$x_{0}$	Number of Parameters	Accuracy (Training)	Accuracy (Validation)	Best Epoch	Epoch Time [s]
64	1,590,148	92.77%	92.17%	30	24
32	399,556	90.00%	92.00%	9	16
16	100,900	92.29%	91.10%	25	15

**Table 8:** Impact of additional attributes on the performance of the model.

Additional Features	Number of Parameters	Accuracy (Training)	Accuracy (Validation)	Best Epoch
**None**	399,556	90.00%	92.00%	9
Asymmetry	399,560	89.96%	91.03%	15
Efficiency	399,560	91.34%	91.90%	61
Fractal dimension	399,560	91.20%	91.17%	23
TAMSD	399,560	90.54%	91.03%	34
All	399,572	83.82%	83.97%	12

**Table 9:** Using autocorrelation function as additional input to the model.

Autocorrelation	Number of Parameters	Accuracy (Training)	Accuracy (Validation)	Best Epoch
**No**	399,556	90.00%	92.00%	9
Yes	401,872	91.55%	91.93%	19

**Table 10:** Different scenarios of selective backprop and their impact on the accuracy of the model.

Scenario	Number of Parameters	Accuracy (Training)	Accuracy (Validation)	Best Epoch	Epoch Time [s]
**None**	399,556	90.00%	92.00%	9	16
98% of cost	399,556	81.81%	90.67%	14	11
50% of cost	399,556	80.57%	90.97%	19	10

**Table 11:** Impact of cost function on the accuracy of the model.

Cost Function	Accuracy (Training)	Accuracy (Validation)	Best Epoch
Cross-entropy	91.91%	91.97%	26
**MSE**	90.00%	92.00%	9

**Table 12:** Accuracy of the model for different choices of the activation function.

Activation Function	Accuracy (Training)	Accuracy (Validation)	Best Epoch
Sigmoid	87.65%	85.13%	10
**ReLU**	90.00%	92.00%	9
LeakReLU	91.50%	91.53%	24
ELU	85.15%	87.20%	3

**Table 13:** Accuracy of the model for different batch sizes.

Batch Size	Accuracy (Training)	Accuracy (Validation)	Best Epoch
256	89.15%	91.63%	7
**512**	90.00%	92.00%	9
1024	94.75%	91.37%	23

**Table 14:** Details of the optimal architecture.

Category	Feature	Value
Architecture	XResNet	Yes
	Dimension	1D
	Depth	3
	Feature map number	32
Modifications	Additional attributes	No
	Autocorrelation	No
	Filtering	No
hyperparameters	Conv. kernel	1×5
	Cost function	MSE
	Activation function	ReLU
	Batch size	512
	Activation threshold	Yes
	Learning rate	0.0003

**Table 15:** Basic performance metrics of the model on test data.

	Accuracy	Precision	Recall	F1 Score
FBM	-	96.98%	98.40%	97.68%
CD	-	91.77%	96.67%	94.16%
DM	-	92.33%	81.87%	86.78%
ND	-	81.76%	85.47%	83.57%
Total/Average	90.6%	90.71%	90.6%	90.55%

**Table 16:** Classification of real data: comparison of our model with the feature based ML method from Ref. [47] (Set A with noise) and the statistical hypothesis testing from Ref. [38,46] (MAX method). “Rec.” and “G Prot.” stand for G protein-coupled receptors and G proteins, respectively. Due to rounding, the numbers may not add up precisely to 100%.

	Our Model		Set A with Noise		MAX Method	
	Rec.	G Prot.	Rec.	G Prot.	Rec.	G Prot.
Subdiffusion	0%	0.6%	25%	34%	21%	24%
Normal diffusion	70%	65%	72%	58%	79%	76%
Superdiffusion	30%	34.4%	1%	6%	0%	1%

## Data Availability

Codes required to generate training datasets may be found at https://github.com/milySW/NNResearchAPI (accessed on 20 May 2021).
